# The Role of Vitamin K Status in Cardiovascular Health: Evidence from Observational and Clinical Studies

**DOI:** 10.1007/s13668-017-0208-8

**Published:** 2017-07-10

**Authors:** A. J. van Ballegooijen, J. W. Beulens

**Affiliations:** 1 Department of Health Sciences, Amsterdam Public Health Research Institute, Vrije Universiteit Amsterdam, De Boelelaan 1085, 1081 HV Amsterdam, the Netherlands; 2 Department of Epidemiology and Biostatistics, Amsterdam Public Health Research Institute, VU University Medical Center, Amsterdam, the Netherlands; 3 Julius Center for Health Sciences and Primary Care, University Medical Center Utrecht, Utrecht, the Netherlands

**Keywords:** Vitamin K, Matrix gla protein, Vascular calcification, Cardiovascular disease

## Abstract

**Purpose of Review:**

Vitamin K is a fat-soluble vitamin required for the activation of several vitamin K-dependent proteins to confer functioning. A growing body of evidence supports that vitamin K has beneficial effects on bone and cardiovascular health. This review summarizes key evidence on vitamin K status as measured by circulating measures and cardiovascular outcomes.

**Recent Findings:**

Overall, observational studies indicate that low vitamin K status as measured by high dephosphorylated uncarboxylated matrix gla protein concentrations plays a potential role in cardiovascular disease development, particularly in high-risk and chronic kidney disease populations. Very few vitamin K intervention trials have been conducted with cardiovascular-related outcomes. A couple of intervention trials studied the effect of the combination of vitamin D + K supplementation, which might have synergistic effects compared to vitamin K supplementation alone.

**Summary:**

Assessing vitamin K status in prospective studies and well-designed randomized trials would provide important insight whether vitamin K is causally related to vascular calcification and cardiovascular disease.

## Introduction

Vitamin K is a fat-soluble vitamin and is mostly known for its function in blood coagulation. Vitamin K was discovered in 1939 by Henrick Dam, who named the molecule vitamin K according to the Danish word for blood clotting *koagulation*.

Vitamin K occurs in our diet in two forms: vitamin K_1_ (phylloquinone) mostly found in green leafy vegetables and vitamin K_2_ (menaquinones) mainly found in animal foods, fermented dairy such as cheese, and natto (fermented soy beans). Vitamin K_2_ includes a range of vitamin K forms and differs from vitamin K_1_ in its side-chain length and degree of saturation. Vitamin K_2_ is the most biologically active form and has a longer half-life than (days vs. hours) vitamin K_1_ [1].

Vitamin K is required as a co-factor in the process of gamma-carboxylation of several vitamin K-dependent proteins turning inactive uncarboxylated proteins into active carboxylated forms to confer functioning. The most well-known vitamin K-dependent proteins are the hepatic coagulation factors prothrombin and factor X. However, other extra-hepatic vitamin K-dependent proteins have been identified as well. Matrix gla protein (MGP) is a small extracellular matrix protein, synthesized in smooth muscle cells, that binds Ca^2+^ ions in the vascular wall and functions as a potent inhibitor of vascular calcification [2]. Vitamin K deficiency results in the synthesis of under-carboxylated, biologically inactive gla proteins—a risk factor for vascular calcification and cardiovascular disease (CVD) [3–6].

Previous studies mostly relied on dietary intake measures of vitamin K such as vitamin K_1_, vitamin K_2_, and natto intake to study the relationship with chronic diseases and CVD [3, 7, 8]. These studies indicated that a high vitamin K intake is associated with improved cardiovascular health. However, self-reported dietary intake is imprecise and inherent to limitations to estimate nutrient intakes [9, 10]. New assay development allows the quantification of various circulating vitamin K status markers such as plasma vitamin K_1_ and K_2_ (menaquinones 4 and 7) and dephosphorylated uncarboxylated MGP (dp-ucMGP) [11]. The purpose of this review is to summarize key evidence on vitamin K status as measured by circulating measures and cardiovascular-related outcomes in humans. Further, novel insights into the combination of vitamin D and vitamin K suggest synergistic effects for cardiovascular health. Vitamin D may preserve vitamin K-dependent protein activity and can thereby contribute to vascular health. Additionally, the interaction with vitamin D is discussed and future research recommendations are given.

## Circulating Vitamin K Status Markers

Circulating nutrient biomarkers are considered more objective measures of nutrient status compared to dietary intake measures and reflect both intake and metabolism. Multiple biomarkers are available to measure vitamin K status, but none of them is robust enough to be considered “gold standard” [12]. Circulating vitamin K_1_ concentrations decrease during vitamin K_1_ depletion and increase with vitamin K_1_ supplementation [13]. An important limitation of the measurement of plasma vitamin K_1_ is that it mainly reflects the intake of the previous days due to its relatively short half-life time of 1–3 h [14].

Menaquinone can be produced by intestinal gut microbiota; however, very little is known about the absorption in the gut and the contribution of the microbiome to vitamin K status [1, 14]. Moreover, the vast majority of the gut menaquinone pool is located in bacterial membranes and is probably not available for absorption; however, data is very limited and a better understanding of how and where menaquinone absorption takes place is urgently needed [14].

Circulating menaquinone concentrations have been rarely studied because specific menaquinones are highly dependent on intake of certain foods, which differs per geographical location. For instance, in a normal Western diet, menaquinone-4 is the only vitamin K_2_ form that is detectable. In a diet without natto intake, menaquinone-7 is often below the limit of detection due to very low concentrations and [11] is only detectable after natto supplementation or menquinone-7 supplementation.

Vitamin K status can also be estimated by measuring the uncarboxylated fractions of certain vitamin K-dependent proteins such as osteocalcin (a marker of bone formation) or MGP, which is the most studied vitamin K-dependent protein in the regulation of vascular calcification. MGP is synthesized by smooth muscle cells in the arterial wall, and higher uncarboxylated concentrations of MGP reflect lower vitamin K status. Since MGP is the main vitamin K status marker of vascular calcification, studies that used osteocalcin concentrations as a marker of vitamin K status with cardiovascular-related outcomes have not been taken into account.

Assays that measure total circulating MGP (regardless of its carboxylation status) have been available for some time [4, 5]. Recently, assays that measure different fractions of MGP in circulation have been developed, of which dephosphorylated uncarboxylated MGP (dp-ucMGP) best reflects vitamin K status [6]. It is considered as a functional marker of bioactivity of both vitamin K_1_ and vitamin K_2_ over multiple weeks to months [15]. Nowadays, dp-ucMGP is available as a fully automated commercial assay, which makes it a feasible marker as routine laboratory assessment in clinical practice. Dp-ucMGP has been proposed as an extra-hepatic vitamin K status marker; however, evidence is currently insufficient to support what levels of dp-ucMGP are required for optimal functioning and more research is needed.

Proteins induced by vitamin K absence factor II (PIVKA-II) uncarboxylated prothrombin are another functional marker of vitamin K status, also known as des-gamma carboxyprothrombin (DCP). PIVKA-II/DCP is detectable in people with deficiency of vitamin K (due to poor nutrition or malabsorption) and in those taking warfarin or other medication that inhibits the action of vitamin K. PIVKA-II/DCP has recently been used in general population studies [16, 17]; however, the commercially available assays have low sensitivity for detecting enough variation in PIVK-II concentrations in healthy populations [12]. The exploration of the biochemistry and physiology of vitamin K biomarkers is ongoing, and relationships with vascular calcification and CVD will be clarified by longitudinal analyses of vitamin K biomarkers in large population-based studies. Possibly, vitamin K status may be estimated more accurately if multiple biomarkers, or biomarkers in combination with dietary intake, are used.

## Cardiovascular-Related Outcomes

Vascular calcification, regardless of its anatomical site, is a strong risk factor for cardiovascular death [18]. Calcification in the vasculature leads to arterial stiffening, elevated systolic pressure, and increased cardiac workload [19]. There is currently no effective treatment available for vascular calcification, and treatment is targeted to relieve symptoms. Therefore, understanding underlying mechanisms driving these processes is urgently needed to lower the burden of vascular calcification and associated health-care costs.

A strong body of evidence from experimental animal models indicates that vitamin K is involved in vascular calcification through carboxylation of MGP [2, 20]. An MGP knock-out model showed extensive calcification in coronary arteries leading to aortic rupture and premature mortality [2]. Further, vitamin K antagonism due to warfarin antagonizes vitamin K-dependent carboxylation of MGP leading to rapid arterial calcification [21]. In addition, a high vitamin K diet is able to reverse aortic calcification after warfarin treatment in rats [22].

The relationship between circulating vitamin K status biomarkers with cardiovascular-related outcomes received growing research interest in the last 5 years. We examined human evidence of circulating vitamin K status and cardiovascular health, with a particular focus on chronic kidney disease (CKD) patients, a group characterized with a disproportional high risk for vascular calcification and CVD death.

## Circulating Vitamin K and CVD-Related Outcomes

A growing amount of studies assessed the cross-sectional relationship between circulating vitamin K status and CVD-related outcomes [17, 23–29] as depicted in Table 1. In two studies from Norway, univariate correlations were found between higher plasma dp-ucMGP and unfavorable echocardiographic measures; however, multivariable statistical analyses were lacking [28, 29]. The study by Dalmeijer et al. [23] showed that higher plasma dp-ucMGP concentrations indicated a higher trend for a higher coronary artery calcification (CAC) score *β* 0.091 (95% confidence interval, −0.01, 0.19) among post-menopausal women. Liabeuf et al. indicated that higher dp-ucMGP was significantly associated with a higher odds of peripheral arterial calcification score: odds ratio 1.88 (1.14, 3.11) [24]. Higher plasma dp-ucMGP was consistently associated with higher carotid-femoral pulse wave velocity (cf-PWV) in populations from different countries [25–27]. In the MESA study, no association between circulating DCP and cardiovascular calcification was observed in healthy participants [17].

**Table 1:** Summary of observational studies of circulating vitamin K status and cardiovascular-related outcomes

Author, year	Country	Study name	Study design	Participants	Vitamin K status exposure	Outcome	Results for highest vs. lowest quantile
Cross-sectional studies							
Ueland, 2010 [28]	Norway	N/A	Cross-sectional	*N* = 147 calcific valvular aortic stenosis patients, age 74 ± 10, 45% female	Plasma dp-cMGP	Echocardiographic measures	Univariate TrP max (mmHg) *r* = 0.23, CI (L/min/m^2^) *r* = −0.31, LVEF (%) *r* = −0.21, nt-proBNP *r* = 0.25. *P* all <0.05
Ueland, 2011 [29]	Norway	N/A	Cross-sectional	179 HF patients and *N* = 33 healthy individuals, age 56 ± 12, 22% female	Plasma dp-ucMGP (nmol/L)	Systolic function, biochemical markers	*N* = 212, univariate correlations, LVEF (%) *r* = −0.32, NT-proBNP (pmol/L) *r* = 0.44 *P* < 0.001
Dalmeijer, 2013 [23]	Netherlands	EPIC-NL	Cross-sectional	*N* = 195, post-menopausal women, age 66.9 ± 5.5	Plasma dp-ucMGP (pmol/L)	CAC score	CAC *β* 0.091 (−0.01, 0.19)
Liabeuf, 2014 [24]	France	DIACART study	Cross-sectional	*N* = 198 type 2 diabetes, men >50 years, women >60 years	Plasma ln dp-ucMGP (pmol/L)	Peripheral arterial calcification score	OR 1.88 (1.14, 3.11)
Pivin, 2015 [25]	Switzerland	SKIPOGH Study	Cross-sectional	*N* = 1001, age 46.5 ± 17.2, 52% female	Plasma dp-ucMGP (nmol/L)	Aortic pulse wave velocity	Aortic PWV *β* 0.186 m/s *P* < 0.001
Mayer, 2016 [26]	Czech Republic	MONICA Study	Cross-sectional	*N* = 1087, age 54.8 ± 13, 53% female	Plasma dp-ucMGP (pmol/L)	Aortic and distal pulse wave velocity	Higher aortic PWV *P* = 0.031
Sardana, 2016 [27]	USA	N/A	Cross-sectional	*N* = 66, type 2 diabetes, age 62 ± 12 years, 9% female	Plasma dp-ucMGP (nmol/L)	Carotid-femoral pulse wave velocity	Higher CF-PWV 0.40 m/s *P* = 0.011
Danziger, 2016 [17]	USA	MESA	Cross-sectional	*N* = 717 weighted cohort, *N* = 104 selected based on AB-index ≥1.4	Circulating DCP ng/mL	Vascular calcification and vascular stiffness measures	DCP not associated with prevalent vascular calcification or stiffness
Case-control studies							
Shea, 2013 [30••]	USA	MESA	Case-control	*N* = 296 extreme CAC progression, *N* = 561 randomly selected Age 64 ± 10, 45% female, 42% White	Plasma vitamin K_1_ (mmol/L)	CAC Agatson score progression	In anti-hypertensive users *N* = 369, OR 2.37 (1.38, 4.09)
Longitudinal studies							
Dalmeijer, 2013 [31•]	Netherlands	EPIC-NL	Longitudinal, 11.2 years follow-up	*N* = 518, type 2 diabetes, age 58.1 ± 7.1, 82% female	Plasma dp-ucMGP (nmol/L)	Incident CVD	SD_increment_ 1.21 (1.06, 1.38) CVD, 1.33 (1.07, 1.65) PAD, 1.75 (1.42, 2.17) HF
Dalmeijer, 2013 [32, 33]	Netherlands	EPIC-NL	Longitudinal, 8.5 years follow-up	*N* = 508 Post-menopausal women, age 56 ± 6	Plasma dp-ucMGP (nmol/L) Plasma vitamin K_1_ (mmol/L)	Coronary calcification and calcified areas	CAC prevalence_SD_ 1.07 (0.99–1.15) Calcified areas RR_SD (4 vs. 0 areas)_ 1.49 (0.95, 2.35) CAC prevalence _T1 vs. T3_ 1.36 (1.02, 1.81) Calcified areas 1.60 (0.65, 3.99)
Van den Heuvel, 2014 [34]	Netherlands	LASA	Longitudinal, 5.6 years follow-up	*n* = 577, age 59.9 years, 56% female	Plasma dp-ucMGP (nmol/L)	Incident CVD	Tertile 3 > 400 pmol/L HR 2.69 (1.09, 6.62)
Dalmeijer, 2014 [35]	Netherlands	EPIC-NL	Case-cohort, 11.5 years follow-up	*N* = 2985, age 49.5 ± 11.8, 75% female	Plasma dp-ucMGP (nmol/L)	Incident CVD and stroke	CVD HR 0.94 (0.79, 1.13), stroke HR 1.09 (0.78, 1.51)
Danziger, 2016 [16]	USA	MESA	Case-cohort, 11 years follow-up	*N* = 709 weighted cohort, *N* = 104 selected based on AB-index ≥1.4	Circulating DCP ng/mL	Ischemic cardiovascular disease	HR per doubling 1.53 (1.09, 2.13)
Liu, 2015 [36]	Belgium	FLEMENGHO	Mendelian randomization study, 14.1 years follow-up	*N* = 2318, age 43.5, 51% female	Plasma dp-ucMGP (nmol/L)	Incident CVD and mortality	Per doubling 1.14 (1.01, 1.28) CVD mortality 0.93 (0.88, 0.99) coronary events
Longitudinal studies among cardiac patients							
Ueland, 2010 [28]	Norway	N/A	Longitudinal, 23 months follow-up	*N* = 147 aortic stenosis patients, age 74 ± 10, 45% female	Plasma dp-ucMGP (pmol/L)	All-cause mortality	HR 4.07 (1.02, 16.22) In non-warfarin users *n* = 118 HR 7.35 (1.59, 34.0)
Ueland, 2011 [29]	Norway	N/A	Longitudinal, 2.9 years follow-up	179 HF patients and *N* = 33 healthy individuals, age 56 ± 12, 22% female	Plasma dp-ucMGP (pmol/L)	Mortality due to HF progression	HR 5.62 (2.05, 15.46)—85th vs. 15th percentile
Mayer, 2014 & 2016 [26, 37•]	Czech Republic	N/A	Longitudinal, 5.6 years follow-up	*N* = 799, prior CVD patients, age 65.1 years, 29% female	Plasma dp-ucMGP (pmol/L)	All-cause mortality, CVD mortality	All-cause HR 1.89 (1.32, 2.72) CVD mortality 1.88 (1.22, 2.90) Q4 vs. Q1

*Dp-ucMGP* dephosphorylated uncarboxylated matrix gla protein, *TrP max* maximum peak tricuspid regurgitation pressure gradient, *CI* cardiac index, *LVEF* left ventricular ejection fraction, *Nt-pro BNP* N-terminal prohormone of brain natriuretic peptide, *OR* odds ratio, *HR* hazard ratio, *N/A* not applicable

In a case-control study from the MESA cohort, lower plasma vitamin K_1_ concentrations were associated with greater coronary artery calcification (CAC) progression in anti-hypertensive medication users (OR 2.37 (1.38, 4.09)), meaning that vitamin K may mediate vascular calcification among high-risk participants [30••].

Also, longitudinal studies assessed the relationship between circulating vitamin K status and coronary calcification or incident CVD [16, 31•, 32–35]. Among type 2 diabetes patients, higher dp-ucMGP was associated with incident CVD hazard ratio (HR) 1.21 (1.06, 1.38) [31•]. Also, in a general older population, higher plasma dp-ucMGP was associated with a higher risk of incident CVD: HR 2.69 (1.09, 6.62) [34]; however, this could not be confirmed in a cohort of middle-age adults: HR 0.94 (0.79, 1.13) [35].

In a cohort of post-menopausal women, higher plasma dp-ucMGP showed a trend for higher coronary calcification risk (RR 1.07 (0.99, 1.15)), but was borderline significant [32]. On the contrary, in the same cohort, higher plasma vitamin K_1_ was not associated with a reduced CAC score but was associated with a higher CAC prevalence ratio, 1.36 (1.02, 1.81) [33].

In a multi-ethnic cohort study, higher circulating DCP was associated with a higher risk of ischemic CVD in a population enriched for ankle-brachial pressure ≥1.4 [16]. In a Flemish population study, Mendelian randomization was applied using genetic variation associated with dp-ucMGP concentrations [36]. Dp-ucMGP correlated significantly with rs2098435, rs4236, and rs2430692, major allele carriers having higher dp-ucMGP concentrations. The instrumental variable analysis did not support a causal association for all-cause and cardiovascular mortality; however, for rs2098435, the HR for coronary events was 0.75 (0.59, 0.96). The authors suggest that the inverse association might be due to inhibition of calcified plaques by active MGP, which is a risk factor in the coronary circulation.

Several prospective studies investigated the relationships between dp-ucMGP concentrations and all-cause mortality among cardiac patients. The studies were performed in Norway and Czech Republic and indicated that higher plasma dp-ucMGP was strongly associated with a higher risk of all-cause mortality [28, 29, 37•]. Further, the study by Mayer et al. indicates that the association is stronger among participants with high B-type natriuretic peptide and dp-ucMGP concentrations for all-cause mortality: HR 2.57 (1.60, 4.10) [26]. This means that high-risk patients may be more prone to the detrimental effects of vitamin K deficiency. In total, growing evidence points out that a low circulating vitamin K status as measured by high dp-ucMGP is related to a higher cardiovascular risk; however, this was not consistent in all studies.

So far, vitamin K intervention trials with hard clinical endpoints are missing. One intervention trial studied the effect of vitamin K vs. placebo on arterial stiffness in healthy post-menopausal women [38]. After 3 years, the beta stiffness index as a measure of mechanical arterial properties decreased significantly in the menquinone-7 group compared to that of the placebo. More studies are clearly needed to investigate whether vitamin K supplementation improves cardiovascular health.

## Chronic Kidney Disease Populations

Vascular calcification is highly prevalent in CKD patients and is a strong predictor of cardiovascular mortality [39, 40]. Vitamin K deficiency is also highly prevalent among CKD populations [41, 42]. In experimental CKD models, vitamin K is key to the susceptibility of vascular calcification [43]. In rats with CKD, the administration of therapeutic doses of vitamin K antagonists or the use of low vitamin K_1_ intake markedly increased the degree of vascular calcification. Further, treatment with high doses of vitamin K increases vitamin K tissue concentrations, attenuates development of calcification, and restores tissue calcium content comparable to that of non-CKD animals [43].

In observational studies among CKD patients, circulating vitamin K is related to cardiovascular-related outcomes as depicted in Table 2. It should be noted that most studies are small (*n* = 40–518) and are conducted among patients that are often restricted in potassium-rich foods that are good sources of vitamin K. PIVKA-II is a good marker for vitamin K deficiency in CKD populations, since it is not affected by kidney function; however, studies are very scarce [46].

**Table 2:** Summary of observational studies of circulating vitamin K status and cardiovascular-related outcomes in chronic kidney disease populations

Author, year	Country	Study design	Participants	Vitamin K status exposure	Outcome	Results for highest vs. lowest quantile
Cross-sectional CKD studies						
Cranenburg, 2009 [44]	Netherlands	Cross-sectional	*N* = 40 hemodialysis patients	Plasma ucMGP (pmol/L)	CAC scores	*β* = 0.004, *P* = 0.02
Delanaye, 2014 [45]	Belgium	Cross-sectional	*N* = 160 hemodialysis patients, age 74 years, 56% female	Plasma dp-ucMGP (pmol/L)	Calcification score	*β* = 0.19 *P* = 0.021
Meuwese, 2015 [46]	Sweden	Cross-sectional	*N* = 97 end-stage renal disease patients, 65% dialysis, age 45.1 years, 63% female	Dp-ucMGP (pmol/L), PIVKA-II (mAU/ml)	Coronary calcification score, arterial stiffness	Not associated with calcification, aortic augmentation pressure *β* 2.2 (−1, 5.4) NS
Thamratnopkoon, 2016 [47]	Thailand	Cross-sectional	*N* = 83 CKD 3–5 patients, age 64.8 years, 44% female	Plasma dp-ucMGP (pmol/L)	Abdominal aorta calcification	OR 1.002 (1.001, 1.004)
Longitudinal CKD studies						
Schurgers, 2010 [48]	France	Longitudinal 2.2 years follow-up	*N* = 107 CKD patients stage 2–5, age 67 years, 40% female	Plasma dp-ucMGP (pmol/L)	All-cause mortality	HR 1.57 (0.67, 3.67)
Schlieper, 2011 [49]	Serbia	Longitudinal 3 years follow-up	*N* = 188 hemodialysis patients	Plasma dp-ucMGP (pmol/L)	All-cause mortality and CVD mortality	HR 2.16 (1.1, 4.3) all-cause mortality, 2.74 (1.2, 6.2) CVD mortality
Keyzer, 2015 [50••]	Netherlands	Longitudinal, 9.8 years follow-up	*N* = 518 kidney transplant recipients	Plasma dp-ucMGP (pmol/L)	All-cause mortality	HR 2.00 (1.20, 3.35), Q4 vs. Q1

*Dp-ucMGP* dephosphorylated uncarboxylated matrix gla protein, *CKD* chronic kidney disease, *NS* non-significant, *OR* odds ratio, *HR* hazard ratio

In cross-sectional studies, higher uncarboxylated MGP [44] and dp-ucMGP [45, 47] were associated with higher calcification scores and prevalent calcification in CKD patients; however, another cross-sectional study could not confirm this [46]. Longitudinal studies from France, Serbia, and the Netherlands studied relationships between plasma dp-ucMGP and mortality risk [48, 49, 50••] among different CKD populations. The study by Schurgers et al. did not observe a significant relationship between dp-ucMGP and all-cause mortality [48], while the study by Schlieper et al. observed a positive relationship with all-cause mortality: HR 2.16 (1.1, 4.3), which was more pronounced for cardiovascular mortality: HR 2.74 (1.2, 6.2) [49]. Among kidney transplant recipients, Keizer et al. observed a strong association between higher dp-ucMGP concentrations and a higher risk of all-cause mortality over 9.8 years of follow-up [50••]. Taken together, in most CKD populations, higher dp-ucMGP concentrations are associated with vascular calcification and all-cause mortality, although this was not consistent in all studies. Future studies would benefit from using long-term CVD outcomes in populations with different stages of kidney disease.

## Interaction with Vitamin D

Vitamin D is a fat-soluble vitamin that can be acquired by ingesting foods such as fatty fish, dairy products, and eggs, but is mainly synthesized by the human skin when exposed to sunlight. Vitamin D is metabolized by the kidney for full biological activity into its most active form 1,25-dihydroxyvitamin D also known as calcitriol. The role of vitamin K in cardiovascular health has mainly been studied in isolation; however, new insights suggest a synergistic effect of vitamin K combined with vitamin D [51–56]. These findings cannot be explained by our current understanding of the biochemical role of vitamin K, but suggest that vitamin D may influence MGP concentrations.

Some animal studies indicate that calcitriol has direct effects on the γ-carboxylase system by stimulating vitamin K-dependent proteins [51–53], which means that the amount of vitamin K-dependent proteins available for carboxylation is vitamin D dependent. This may lead to higher circulating levels of under-carboxylated MGP and calcium deposition in the vasculature, which could further increase the risk of vascular calcification and CVD. In vitro studies also support the concept of a synergistic effect of vitamin K and vitamin D. These studies found that the matrix gla protein gene promoter contains a vitamin D response element, capable of a two- to threefold enhanced matrix gla protein expression after vitamin D binding [54–56]. The effect of vitamin D combined with vitamin K on dp-ucMGP is therefore expected to be larger than that of solely vitamin K; however, this should be further explored.

## Clinical Trials with Combined Vitamin D and K Supplementation

So far, two human intervention studies in healthy populations have investigated the combined effect of vitamins D and K on vascular function and calcification [57, 58]. In post-menopausal women after 3 years of supplementation (1000 μg/day vitamin K_1_ + 320 IU vitamin D), the vitamin D + K group maintained vessel wall characteristics of the carotid artery, whereas the control group and the vitamin D-only group significantly worsened over 3 years of follow-up [57]. However, vitamin K status was not measured as a marker of compliance to investigate what occurs following supplementation. Further, in a 3-year, double-blind, randomized controlled trial in older men and women free of clinical CVD, daily supplemental vitamin K in amounts achievable by high dietary intake of green, leafy vegetables (500 μg/day) combined with 600 mg calcium carbonate and 10 μg (400 IU) vitamin D did not result in lower CAC progression compared to the calcium + vitamin D group. In a subgroup analysis of participants who were ≥85% adherent to supplementation, there was less CAC progression in the vitamin K + calcium and vitamin D group than in the calcium and vitamin D group alone [58]. These data are hypothesis generating, and further studies are warranted to clarify the mechanism.

Two trials studied the effect of vitamin D vs. vitamin D + K in non-dialyzed CKD patients on vascular calcification and cardiovascular risk factors for 9 months [59, 60]. In 42 CKD patients, the increase in common carotid intima-media thickness (CCA-IMT) was significantly lower in the K (90 μg menaquinone-7) + D (10 μg vitamin D) compared with the D-only group after 9 months [59]. Another small trial (*n* = 38) from the same research group did not show differences between the D vs. D + K group on cardiovascular risk markers [60]. These few studies show some potential for the combined effect of vitamin D + K vs. D alone on cardiovascular-related outcomes. It should be noted that very few clinical studies have been conducted and vitamin D and K are combined with different micronutrients making it difficult to solely pinpoint the effect to vitamin D + K.

## Recommendation for Future Research

- Study the effect of different vitamin K forms in relation to cardiovascular-related outcomes
- Define the clinical cutoff value for various vitamin K status markers and define vitamin K deficiency
- Deepen the knowledge on the interaction between vitamins D and K and cardiovascular-related outcomes

## Conclusions

Overall, observational studies indicate that vitamin K has a potential role in cardiovascular health particularly in high-risk and chronic kidney disease populations. Vitamin K intervention trials with subclinical cardiovascular endpoints are scarce. Most clinical studies investigated the combination of vitamin D + K supplementation, which might have synergistic effects compared to vitamin K supplementation. Vitamin D may preserve vitamin K-dependent protein activity and can thereby contribute to vascular health. Assessing vitamin K status using multiple biomarkers in prospective studies and well-designed randomized trials would provide important insight whether vitamin K is causally related to vascular calcification and CVD.
